# Oral Administration of Resveratrol Alleviates Osteoarthritis Pathology in C57BL/6J Mice Model Induced by a High-Fat Diet

**DOI:** 10.1155/2017/7659023

**Published:** 2017-01-29

**Authors:** Mengqi Jiang, Xingyao Li, Xiaolu Yu, Xudan Liu, Xiaolei Xu, Jianyi He, Hailun Gu, Li Liu

**Affiliations:** ^1^Department of Nutrition and Food Hygiene, School of Public Health, China Medical University, No. 77 Puhe Road, Shenyang North New Area, Shenyang, Liaoning 110122, China; ^2^Department of Nutrition and Food Hygiene, School of Public Health, Beihua University, No. 3999 Binjiang East Road, Jilin City, Jilin 132013, China; ^3^Department of Orthopedics, Shengjing Hospital, China Medical University, No. 36 Sanhao Street, Heping District, Shenyang, Liaoning 110004, China

## Abstract

Obesity has been associated with osteoarthritis (OA) due to increased mass and metabolic factors which are independent of the biomechanical contribution to joint load. Resveratrol, a natural polyphenolic compound, exerts protective effects on OA through its anti-inflammatory property. However, the mechanism of resveratrol on obesity-related OA is unclear. To investigate the effect and possible mechanism of oral resveratrol on obesity-related OA, we fed C57BL/6J mice with a high-fat diet (HFD) for 16 weeks to establish obesity-related OA model; then two doses (22.5 mg/kg and 45 mg/kg) of resveratrol were given by gavage for additional 12 weeks. Mice with HFD significantly increased body weights compared to the control mice, while resveratrol treatment did not cause obvious weight loss. Histological assessments showed that resveratrol at 45 mg/kg significantly improved OA symptoms. Levels of serum IL-1*β* and leptin were decreased by resveratrol treatment and positively correlated with Mankin scores. Moreover, resveratrol significantly inhibited the expression of TLR4 and TRAF6 in cartilage. These results suggest that HFD induced obesity can lead to the occurrence of OA, and resveratrol may alleviate OA pathology by decreasing the levels of systematic inflammation and/or inhibiting TLR4 signaling pathway in cartilage. Thus, resveratrol might be a promising therapeutic treatment for obesity-related OA.

## 1. Introduction

Osteoarthritis (OA) is the most common chronic joint disease which is due to degenerative changes of the joint. It is generally regarded as a major cause of disability in elderly population. Although the pathogenesis of OA is not fully established, obesity is considered as one of the strongest factors of OA development [1]. The prevalence of knee OA in obese people is 5.27 times higher than that with normal weight [2]. Weight gain changes the joint load and damages the joints, but the inflammatory and metabolic characteristics of the obesity also affect the health of the joints [3]. Different mechanisms may drive different phenotypes of OA, which may require different therapeutic approaches [4]. Recently, metabolic OA which is a subtype of OA has been described as a disorder, which displays a unique OA path that is potentially independent of joint load of biomechanical contributions [5]. Obesity is associated with low-grade systemic inflammation [6] and metabolic factors that link obesity and OA. Therefore, figuring out the mechanism of obesity-related OA is necessary for the treatment of metabolic OA therapy.

Diet-induced obesity can simulate the occurrence of human obesity. The relationship between high-fat diet (HFD) induced obesity and the development of OA has been studied in several animal models [7–10]. It was found that the occurrence of knee OA was twice as C57BL/6 mice on a HFD compared to that in the control group [11]. HFD induced obese C57BL/6 mice manifested inflammation response in adipose tissue and secrete various inflammatory mediators [12] which suggests that HFD fed C57BL/6 mice constitute a suitable model to study obesity-related metabolic OA.

Toll-like receptor 4 (TLR4) is a member of toll-like receptors, the expression of which in cartilage is increased throughout the process of joint degenerative changes [13–15]. Furthermore, TLR4 has been shown to be increased in the injured areas of cartilage in patients with OA [16]. These changes are attributed to their mediated proinflammatory response [17, 18]. It also contributes to the link between inflammation and HFD induced obesity. TLR4 can be activated by saturated fatty acid [19] and recruits the MyD88, which in turn recruits interleukin-1 receptor-associated kinase 1 (IRAK1) and 4 (IRAK4) as a complex to activate tumor necrosis factor receptor-associated factor 6 (TRAF6) and nuclear factor-*κ*B (NF-*κ*B) subsequently to regulate proinflammatory gene expression [20], and recent study has also revealed that saturated fatty acid activates the MyD88 independent signaling pathway to mediate inflammatory cytokine production [21]. Although TLR4 signaling pathway has been studied in chondrocytes and cartilage [15, 22, 23], the role in obesity-related OA is not fully understood.

Current medical treatment strategies for OA are focused on pain remission and symptom control rather than disease modification. These pharmaceutical treatments are limited and can have inevitable side effects [24, 25]. Resveratrol belongs to a large group of biologically active substances found in grape skin and red wine at high concentrations. It is important to note that the effects of resveratrol are mainly observed in animals fed a HFD diet, implying that resveratrol is particularly potent in reversing early stage of metabolic disorders [26]. Recently resveratrol has also been described as a treatment for OA through its anti-inflammatory, antioxidant [27], and antiapoptotic properties [28–31]. However, the mechanisms of resveratrol on obesity-related OA have rarely been studied. In the obese state, hypertrophic white fat tissue releases several proinflammatory mediators and adipokines in blood such as interleukin-1*β* (IL-1*β*), leptin, and adiponectin which may participate in cartilage alteration in obese patients [32, 33]. Resveratrol may exert systemic anti-OA effects by decreasing these cytokines. Previously, we demonstrated that resveratrol can prevent OA via the TLR4/MyD88/NF-*κ*B signaling pathway on human osteoarthritic chondrocytes in vitro [22]. In addition, we also found that supplement with both resveratrol and HFD for 12 weeks could alleviate OA progression in C57BL/6J mice [34] by preventing the degradation of type II collagen and chondrocytes apoptosis. However, whether the effect of oral resveratrol on obesity-related OA was associated with TLR4 signaling pathway is still obscure.

Thus, the aim of our study was to investigate the effects and potential mechanism of oral resveratrol on obesity-related OA pathogenesis. To address this, we first employed C57BL/6J mice fed with HFD to establish an OA model and observed the effects of resveratrol treatment on OA mice. Then, we further investigated whether resveratrol affected obesity-related OA by reducing systematic inflammation and/or inhibiting TLR4 signaling pathway in cartilage.

## 2. Material and Methods

### 2.1. Animals and Treatments

Sixty-four male C57BL/6J mice were obtained from HFK bioscience Co., Ltd. (Beijing, China). At the age of 7 weeks, animals were randomized to either the HFD group (*n* = 47) which were fed a diet containing 58% of its energy derived from fat or the control group (CON, *n* = 17) which were fed a standard chow diet containing 10% of its energy derived from fat. Body weight and food intake were measured every week. After 16 weeks, two mice from CON and HFD group were picked up, respectively, for histological examination to evaluate the establishment of mice OA model induced by HFD. The remaining animals of HFD group were randomly distributed in three groups. One of the groups remained on feeding simple high-fat diet (HFD-S, *n* = 15) while the other two were, respectively, given 22.5 mg/kg (RES22.5, *n* = 15) or 45 mg/kg (RES45, *n* = 15) of resveratrol (Guanyu Biotech, Xi'an, Shanxi, China) diluted in 0.5% carboxymethylcellulose sodium (CMC, diluted in 0.9% normal saline; Sinopharm Chemical Reagent Co., Ltd., Shenyang, Liaoning, China) by oral gavage. At the same time, the CON and HFD-S were also given 0.5% CMC by oral gavage. All of the animals were allowed for active unlimited and free to take food and water with weekly weighing for a total of 12 weeks. All animal procedures were approved by the local Institutional Animal Care Ethics Committee for animal studies at China Medical University.

### 2.2. Histological Examination

At the end of 28 weeks after the initiation of experiment, all animals were sacrificed. Whole knee joints of the mice were fixed in 4% paraformaldehyde at PH 7.4 for 1 d, decalcified for 2 months using 10% EDTA-Na_2_, dehydrated, and embedded in paraffin for histologic analysis. The specimens were serially sectioned at a thickness of 6 *μ*m. The sections were dyed with Safranin O (Tianjin Guangfu Fine Chemical Research Institute), hematoxylin and eosin (H & E) (Tianjin Guangfu Fine Chemical Research Institute, Tianjin, China), or Safranin O/Fast Green (Sinopharm Chemical Reagent Co., Ltd., Shanghai, China) and were examined microscopically. All sections were observed and evaluated by two researchers. Histological examinations of cartilage were measured by modified Mankin scores [35]. The grading system included four categories: cartilage structure (6 points), cartilage cells (3 points), staining (4 points), and tidemark integrity (1 point).

### 2.3. ELISA Assay

Blood samples were collected from the abdominal aorta and centrifuged at 1000 ×g for 15 min and stored at −80°C. The levels of IL-1*β*, leptin, and adiponectin in serum were detected using ELISA kits (Boster Biotechnology, Wuhan, China). All experimental protocols were performed in accordance with the manufacturers' instructions.

### 2.4. RNA Extraction and Real-Time RT-PCR

Total RNA of cartilage was isolated using RNAiso plus (TaKaRa, Dalian, China) in accordance with the manufacturer's instructions. The purity and quantity of extracted RNA were measured and total RNA was reverse-transcribed into single-stranded cDNA. Real-time quantitative PCR was performed by PCR detection system (ABI, Carlsbad, CA, USA). Each PCR reaction mixture contained 0.8 *μ*L of forward and reverse primers, 10 *μ*L of 2x SYBR Green Master Mix (TaKaRa, Dalian, China), 2 *μ*L of cDNA, and 0.4 *μ*L of Rox Reference DyeII (50x) (TaKaRa, Dalian, China). The reaction mixtures were denaturalized at 95°C for 1 min, followed by 40 cycles of 95°C for 5 s and 60°C for 34 s. TLR4 and TRAF6 mRNA levels were normalized to *β*-actin levels (2^−ΔΔCt^ Method). The sequences of the primers (Sangon Biotech, Shanghai, China) used are listed 5′ to 3′ as follows: *β*-actin (171 bp) (F) 5′-CATCCGTAAAGACCTCTATGCCAAC-3′, (R) 5′-ATGGAGCCACCGATCCACA-3′; TLR4 (115 bp) (F) 5′-TCAGAGCCGTTGGTGTATCTT-3′, (R) 5′-CCTCAGCAGGGACTTCTCAA-3′; TRAF6 (110 bp) (F) 5′-GCCGAAATGGAAGCACAG-3′, (R) 5′-GGGCTATGGATGACAACAGG-3′.

### 2.5. Western Blot Analysis

For Western blot analysis, cartilage was separated and prepared before homogenization in RIPA buffer containing PMSF (DingguoChangsheng Biotechnology, Beijing, China). Tissue homogenates were centrifuged (12,000 ×g, 15 min, 4°C) and the supernatants were stored at −80°C until use. The concentrations of total proteins were determined using a BCA kit (Thermo Fisher Scientific, San Jose, CA, USA). Extracted proteins (30 *μ*g/lane) were separated by 8% sodium dodecyl sulfate polyacrylamide gel (SDS-PAGE) electrophoresis and then the separated proteins were transferred onto PVDF membranes. The membranes were then blocked with 5% skimmed milk in TBS-T (20 mM Tris-HCl pH 7.6, 150 mM NaCl, and 0.1% Tween-20) for 2 h at room temperature. After washing with TBS-T, the samples were incubated with the following primary antibodies: *β*-actin (1 : 500, Santa Cruz, CA, USA), TRAF6 (1 : 100, Santa Cruz), and TLR4 (1 : 500, Santa Cruz) in TBS-T containing 3% BSA overnight at 4°C. The blots were washed and incubated with HRP-conjugated secondary antibody (1 : 5000 dilution, DingguoChangsheng Biotechnology, Beijing, China) in TBS-T containing 1% BSA for 1 h at room temperature. After washing with TBS-T, signals were detected using chemiluminescent detection system (Gel Image System Ver.4.00, California, USA). Band quantification was done using the Scion Image 4.0 software (Scion Corporation, Frederick, MD, USA).

### 2.6. Statistical Analysis

All data were presented as means ± SEM. Data were analyzed using one-way analysis of variance (ANOVA) and the statistical analysis was performed using SPSS statistical software (SPSS 13.0 software, SPSS Inc., Chicago, IL, USA). Pearson linear regression was used to determine the degree of association between the cytokines and Mankin scores. Statistical significance was defined as *P* < 0.05.

## 3. Results

### 3.1. Resveratrol Treatment Had No Effect on Body Weight in Obese Mice Fed a HFD

**Figure 1:**
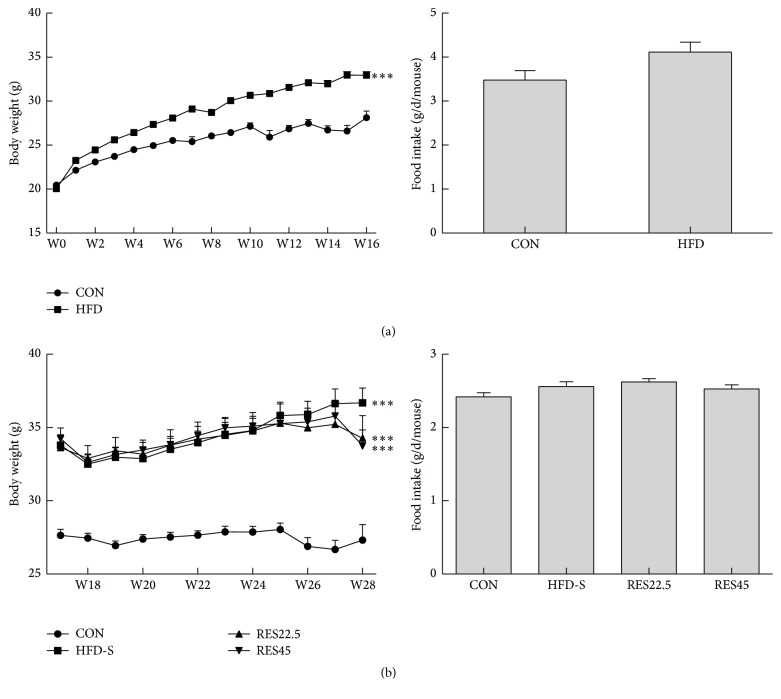
Body weight and food intake. (a) Body weight and food intake during 1–16 weeks in mice fed with HFD (CON, *n* = 17; HFD, *n* = 47). (b) Body weight and food intake during 17–28 weeks after resveratrol treatment in mice fed with HFD (CON, HFD-S, RES22.5, and RES45, *n* = 15). One-way ANOVA was used to test for statistical significance. Data were expressed as the mean ± SEM. ^*∗∗∗*^*P* < 0.001 versus the CON group.

Mice in HFD were significantly heavier than CON from week 2 to week 16 (Figure 1(a)). From week 17 to week 28, body weight of HFD-S and RES groups had been higher than that of CON. Although RES groups slightly lost weight at week 28, there is no statistical difference compared to HFD-S (Figure 1(b)). Meanwhile, the food intake had shown no statistical difference throughout the whole experiment (Figure 1).

### 3.2. Establishment of OA Model in Mice Fed a HFD and Resveratrol Treatment Prevented OA Progression

**Figure 2:**
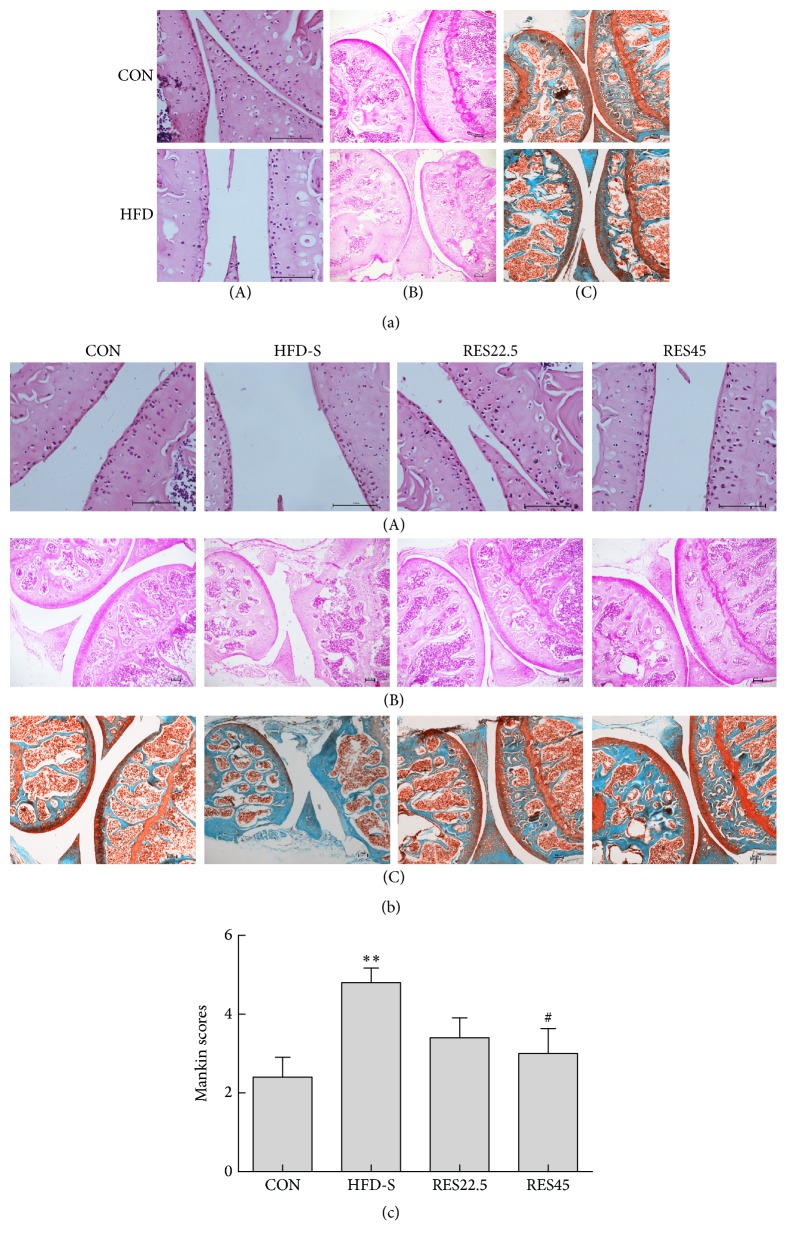
Oral resveratrol prevents articular cartilage damage in a HFD induced OA model. (a) Articular staining of mice fed with HFD at the end of 16 weeks (CON and HFD, *n* = 2). (b) Articular staining of mice after resveratrol treatment at the end of 28 weeks (*n* = 5 per group); (A) H & E (original magnification, 200x); (B) Safranin O (original magnification, 40x); and (C) Safranin O/Fast Green (original magnification, 40x). (c) Modified Mankin scores. Scale bars = 100 *μ*m. One-way ANOVA was used to test for statistical significance. Data were expressed as the mean ± SEM. ^*∗∗*^*P* < 0.01, versus the CON group; ^#^*P* < 0.05 versus the HFD-S group.

Sections stained at week 16 showed that the cartilage thickness was reduced, the arrangement of chondrocyte was disordered, and the cartilage structure exhibited mild damage in HFD group (Figure 2(a)). These results demonstrated the successful establishment of HFD induced knee joint OA model. Moreover, the features of OA were observed more apparently in the HFD-S according to the sections stained at week 28, including the reduction of cells in the knee joints, loss of tide lines, and the damage of articular cartilage in the medial tibial plateaus. Conversely, all these histological manifestations had been improved in resveratrol treatment groups (Figure 2(b)).

A modified Mankin scoring system was used to evaluate the severity of OA. Remarkably, the joints from HFD-S mice showed higher OA scores compared with the CON. After treating with resveratrol, the Mankin scores of two doses were both lower than HFD-S, while the RES45 was more significant (Figure 2(c)). It was consistent with the histological manifestation.

### 3.3. Resveratrol Decreases Levels of Serum IL-1*β* and Leptin

**Figure 3:**
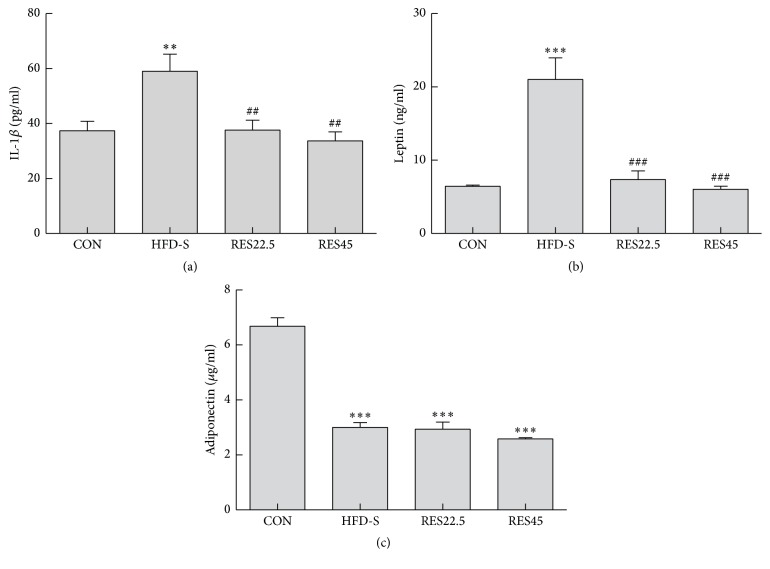
Serum cytokine concentrations of IL-1*β* (a), leptin (b), and adiponectin (c). *n* = 5 mice per group. One-way ANOVA was used to test for statistical significance. Data were expressed as the mean ± SEM. ^*∗∗*^*P* < 0.01 and ^*∗∗∗*^*P* < 0.001 versus the CON group; ^##^*P* < 0.01 and ^###^*P* < 0.001 versus the HFD-S group.

It was reported that there are many cytokines associated with obesity-related OA. Here, we tested the effects of resveratrol on IL-1*β*, leptin, and adiponectin. As shown in Figures 3(a) and 3(b), HFD increased leptin and IL-1*β* levels by 59% and 2.5 times, respectively. Additionally, in RES22.5 and RES45 mice, resveratrol treatment significantly decreased leptin and IL-1*β* levels versus HFD-S. Adiponectin in all HFD fed mice revealed significant differences in comparison to the CON, yet with no apparent differences among the three groups (Figure 3(c)). It is worth mentioning that although there was no difference between two doses, RES45 exerted a better anti-OA efficacy (Figures 3(a), 3(b), and 3(c)).

### 3.4. Association between Body Weight, IL-1*β*, Leptin, and Adiponectin Levels and Mankin Scores

**Figure 4:**
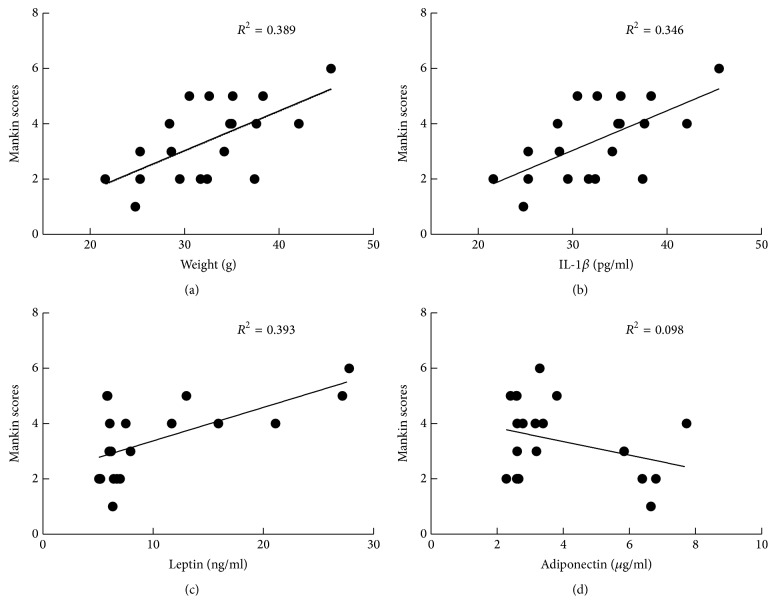
Correlation analysis between body weight, IL-1*β*, leptin, adiponectin levels, and Mankin scores. Pearson's correlation coefficient was calculated for each pair of parameters: (a) body weight and Mankin scores, *R*^2^ = 0.389, *P* = 0.003; (b) serum IL-1*β* levels and Mankin scores, *R*^2^ = 0.346, *P* = 0.006; (c) serum leptin levels and Mankin scores, *R*^2^ = 0.393, *P* = 0.003; (d) serum adiponectin levels and Mankin scores, *R*^2^ = 0.098, *P* = 0.179. Each point on the graph represented a single mouse. *n* = 5 mice per group.

In order to investigate the potential relationship between biomechanical factors, metabolic factors, and OA, we performed a correlation analysis between body weight (Figure 4(a)), IL-1*β* (Figure 4(b)), leptin (Figure 4(c)), and adiponectin (Figure 4(d)) levels and Mankin scores. Results of Pearson correlation coefficients showed that body weight, serum IL-1*β*, and leptin, but not adiponectin, were significantly associated with Mankin scores.

### 3.5. Resveratrol Inhibits TLR4 Signaling Pathway in Articular Cartilage of OA Mice Induced by HFD

**Figure 5:**
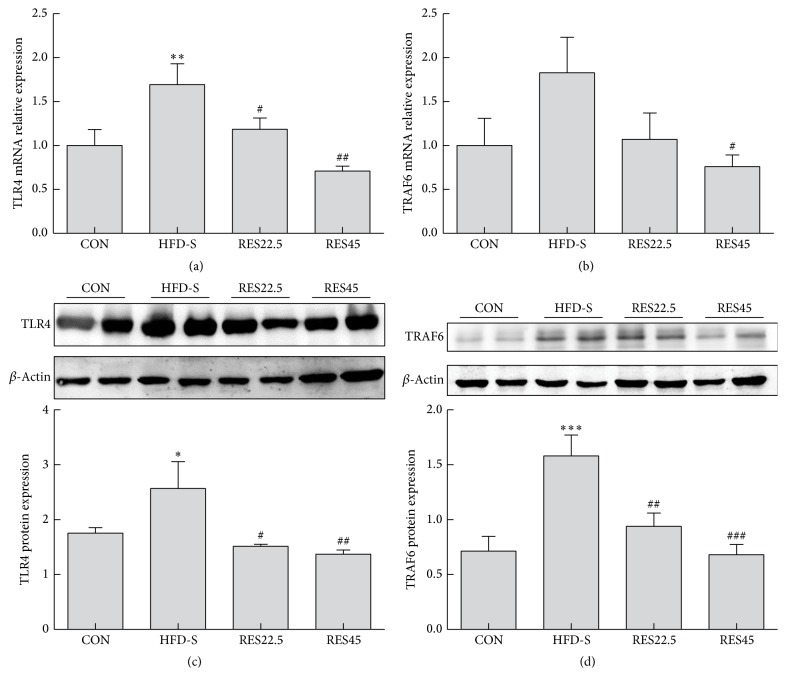
TLR4/TRAF6 mRNA and protein expression. The relative expression levels of TLR4 mRNA (a) and TRAF6 mRNA (b) were determined by real-time RT-PCR. Whole cell protein concentrations were determined and the relative amount of TLR4 (c) and TRAF6 (d) was assessed by Western blot analysis. *n* = 5 mice per group. One-way ANOVA was used to test for statistical significance. Data were expressed as the mean ± SEM. ^*∗*^*P* < 0.05, ^*∗∗*^*P* < 0.01, and ^*∗∗∗*^*P* < 0.001 versus the CON group; ^#^*P* < 0.05, ^##^*P* < 0.01, and ^###^*P* < 0.001 versus the HFD-S group.

In order to investigate whether oral resveratrol inhibits TLR4 signaling pathway in articular cartilage of OA mice, we examined the expression of TLR4 and TRAF6 mRNA and protein. According to the results of real-time RT-PCR and Western blot analysis, the HFD-S trend toward higher expression of TLR4 versus CON, while the expression in resveratrol-treated groups was downregulated (Figures 5(a) and 5(c)). In the gene expression of TRAF6, only RES45 was downregulated compared with that in HFD-S (Figure 5(b)). Consistent with TLR4, the protein expression of TRAF6 in HFD-S is higher than CON, and the expression in resveratrol-treated groups was suppressed (Figure 5(d)). Although there was no statistical difference between two doses of resveratrol, the mRNA expression of TLR4 and TRAF6 in RES45 group is lower than RES22.5 group (TLR4, 40%; TRAF6, 29%) and the protein expression of TLR4 and TRAF6 is also decreased in RES45 group compared with RES22.5 group (TLR4, 10%; TRAF6, 27%).

## 4. Discussion

To establish obesity-related OA model, we fed the mice with HFD for 16 weeks and it was found that HFD could induce obesity and OA, thereby demonstrating successful recapitulation of obesity-related OA. This result is consistent with several animal studies which have shown that HFD increases the severity of OA [36–38]. After OA model establishment, obesity-related OA mice were given by gavage two doses of resveratrol. The results showed that resveratrol could prevent OA progression. This effect was not attributed to body weight loss because resveratrol-treated mice maintained the same body weight as control mice, indicating that resveratrol alleviated OA not only through the mechanical factors. Indeed, the effect of resveratrol on body weight is controversial. Ikuta and colleagues reported that resveratrol reduced obesity in mice that received a HFD [39], and Lagouge et al. showed resveratrol treatment protected mice against diet-induced-obesity by reducing body fat and white adipose tissue [40]. In our previous study, we also found resveratrol has protective effect on OA partly by reducing body weight [34], while, on the contrary, several studies did not observe the same effect [41, 42]. The discrepancy of these results may be due to different dosage of administration, animal species, and initial time of resveratrol supplementation. Resveratrol may exert significant antiobesity effect in mice which were fed both HFD and resveratrol simultaneously but present inconspicuous role once the mice had been induced with obesity by a HFD. In the present study, excessive weight gain contributed to OA development, whereas the anti-OA effects of resveratrol in obese mice may be independent of biomechanical overloading reduction.

Excess weight gain increases the mechanical loads on the joint via physical activity, which most commonly occurs on knee and hip joints. However, there is also an increase trend of OA in non-weight bearing joints, such as hand and temporomandibular joints [43] which have indicated that the connection between overweight and OA might also occur through systemic inflammation. Mice lacking production of functional leptin or lacking functional leptin receptors develop extreme obese phenotypes without increased incidence of knee osteoarthritis which suggest that weight alone may not be a risk factor for joint degeneration [44]. Among obese women, leptin was associated with 28% greater odds of having knee OA (OR = 1.28). Although it was statistically significant (OR = 1.04), among nonobese women, the association between leptin levels and knee OA was less which further indicated that leptin levels were strongly associated with obesity-related OA [45]. Consistent with this, in the present study, a positive correlation between leptin and Mankin scores was found, implying that OA severity had a significant relationship with leptin levels. After treating with resveratrol, it provided protective effects on arthritic changes of the joint and reduced leptin levels. In addition, we also found that HFD reduced the level of adiponectin while resveratrol had no effect on it. The influence of adiponectin on OA is not fully understood, and some evidence suggests that adiponectin has a positive association with cartilage destruction in OA [46]. Nevertheless, adiponectin may prevent OA progression by favoring an anti-inflammatory phenotype in macrophages [47]. Our present findings indicated that the anti-OA effect of resveratrol was not significantly associated with adiponectin levels.

**Figure 6:**
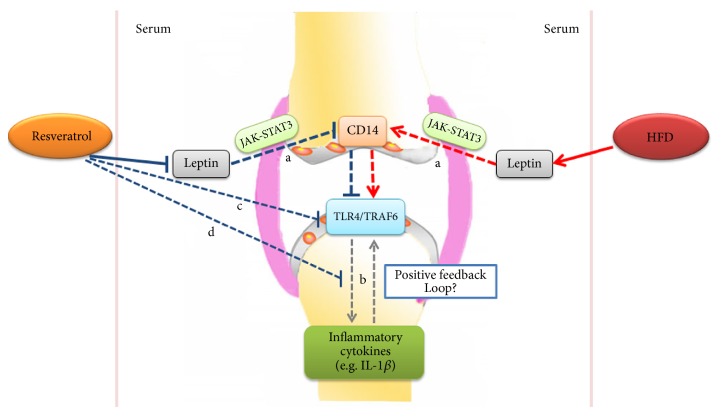
Schematic describing the effects and proposed response on TLR4 signaling pathway and inflammatory cytokines resulting from resveratrol treatment and HFD. HFD stimulated serum leptin level and decreased with resveratrol treatment. Leptin may upregulate CD14 via the JAK-STAT3 signaling pathway and activate CD14-dependent TLR4 pathway to induce an inflammatory response (a, dashed line). A positive feedback loop may exist between IL-1*β* and TLR4 pathway (b, dashed line), and resveratrol-mediated direct (c, dashed line) or indirect (d, dashed line) inhibition of TLR4 expression in cartilage could block the IL-1*β*-TLR4 pathway feedback loop.

TLR4 is a member of the TLR family, potentially related to the onset and progression of OA. Here the elevated expression of TLR4 and its downstream signaling molecule TRAF6 in cartilage of mice fed a HFD was exhibited. This result is in agreement with several researches showing that HFD increases the expression of TLR4 [48–50]. A previous study demonstrated that IL-1*β* induced inflammatory response via TLR4 signaling pathway on human osteoarthritic chondrocytes [22]. Interestingly, an increased level of serum IL-1*β* was also observed here. Excessive serum inflammatory cytokines (IL-1*β*, leptin, etc.) could infiltrate into synovial fluid [51] and thereby resulted in local inflammation in cartilage. These results indicated that a positive feedback loop may exist between IL-1*β* and TLR4 pathway. It has been suggested that resveratrol could reduce the expression of IL-1*β* in human chondrocytes and rabbit cartilage [22, 52]. Moreover, another study has shown that resveratrol reversed significantly IL-1*β*-reduced cell proliferation and blocked pro-IL-1*β* and IL-1*β* synthesis in chondrocytes [28]. Therefore, we proposed that administration of resveratrol may break the loop and reduce the level of IL-1*β* and expression of TLR4 and TRAF6 and significantly mitigated the inflammatory response. In addition, we also found that HFD stimulated serum leptin level and decreased with resveratrol treatment. Leptin is associated with the pathogenesis of OA via the JAK-STAT3 signaling pathway [53]. In nonalcoholic steatohepatitis, leptin can upregulate CD14 expression through JAK-STAT3 signaling pathway [54] and it has been demonstrated that CD14 can activate CD14-dependent TLR4 pathway to induce an inflammatory response [55]. Thereby, we consider that leptin may activate TLR4/TRAF6 signaling pathway via CD14 to promote the secretion of inflammatory cytokines such as IL-1*β* and resveratrol treatment decreased serum leptin level to suppressed TLR4 and TRAF6 expression in cartilage and reduced serum IL-1*β* level (Figure 6).

Whether the leptin plays an initial key role in HFD induced OA still needs to be further investigated. In our present study, the direct mechanism of leptin induced TLR4 signaling pathway was not detected; thus, our following research will focus on the role of leptin in OA pathology and clarifying the cross-talk between leptin and TLR4 signaling pathway.

## 5. Conclusion

We conclude that HFD induced obesity in C57BL/6 mice can lead to the occurrence of OA, and oral resveratrol may alleviate OA pathology by decreasing the levels of cytokines and/or through inhibiting TLR4/TRAF6 signaling pathways. Considering these results, resveratrol could potentially be developed as a promising therapeutic treatment for OA.
